# Actin dynamics in host–pathogen interaction

**DOI:** 10.1002/1873-3468.13173

**Published:** 2018-07-05

**Authors:** Theresia E. B. Stradal, Mario Schelhaas

**Affiliations:** ^1^ Department of Cell Biology Helmholtz Centre for Infection Research (HZI) Braunschweig Germany; ^2^ Institute of Cellular Virology ZMBE University of Münster Germany

**Keywords:** actin dynamics, bacterial invasion, host–pathogen interaction, viral entry, virulence factors

## Abstract

The actin cytoskeleton and Rho GTPase signaling to actin assembly are prime targets of bacterial and viral pathogens, simply because actin is involved in all motile and membrane remodeling processes, such as phagocytosis, macropinocytosis, endocytosis, exocytosis, vesicular trafficking and membrane fusion events, motility, and last but not least, autophagy. This article aims at providing an overview of the most prominent pathogen‐induced or ‐hijacked actin structures, and an outlook on how future research might uncover additional, equally sophisticated interactions.

## Abbreviations

**EHEC**, Enterohemorrhagic *E. coli*

**EPEC**, Enteropathogenic *E. coli*

**GAP**, GTPase‐activating protein

**GDI**, guanine nucleotide dissociation inhibitor

**GEF**, guanine nucleotide exchange factor

**NPF**, nucleation promoting factor

**T3SS**, type III secretion system

## Cellular actin assemblies

The shape of cells, their movement, phagocytosis, intercellular communication, endo‐ and exocytosis as well as the distribution of organelles all depend on dynamic reorganizations of the actin cytoskeleton. Actin exists in the cell in two distinct forms: globular actin (G‐actin) monomers and filamentous actin (F‐actin) polymers. The rearrangement of cellular actin structures is a dynamic, often fast process driven by continuous assembly, disassembly and/or reassembly of actin filaments. This turnover is controlled by multiple factors including major, ubiquitously operating machines, representatives of which are found in all eukaryotes.

### Molecular basis of actin polymerization

**Figure 1:**
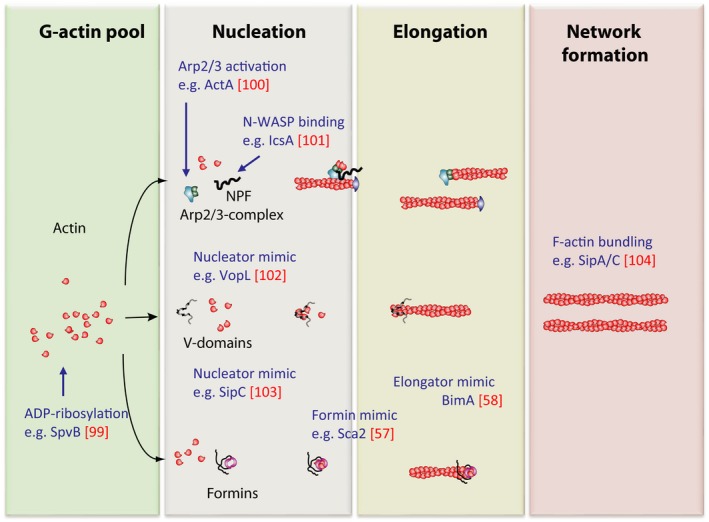
Molecular mechanisms of actin filament assembly and their targeting by virulence factors. Actin filament turnover is tightly regulated by catalytic nanomachines and their cofactors (for details, see text or Ref. 12). Assembly of F‐actin is manipulated at virtually every level by bacterial virulence factors. The columns mark the phases of F‐actin production and the virulence actors (blue) are placed about where they affect filament turnover. The factors depicted are only few examples and the list is far from being complete. Nonetheless, for the future, we expect many more virulence factors and/or mechanisms to be identified that affect these and other steps of dynamic actin turnover such as severing or capping. Note that the molecular mechanisms evolved by bacteria to nucleate/elongate actin are not identical, but at best similar to those of the host cell as drafted in the chart.

The first step in making a filament from G‐actin monomers is the so‐called nucleation, driven by tightly regulated catalytic molecular machines like Arp2/3 complex or members of the formin family of proteins. A schematic overview of the most prominent mechanisms of actin assembly (along with exemplary virulence factors targeting them, see also below) is given in Fig. 1. It is becoming increasingly clear that these and similar machines come as multicomponent complexes, which generate F‐actin in response to signals that are transferred onto these machines foremost by Rho‐GTPases (see below and Refs 1, 2, 3). In case of Arp2/3 complex, an additional class of proteins or protein complexes, namely the so‐called nucleation promoting factors (NPFs) operate as essential intermediates for the activation of actin assembly. Activation of Arp2/3 complex by these NPFs leads to the formation of branched actin networks. Signal‐dependent ignition of any of these machines, therefore, results in the spatiotemporally restricted generation of F‐actin on cellular membranes.

**Figure 2:**
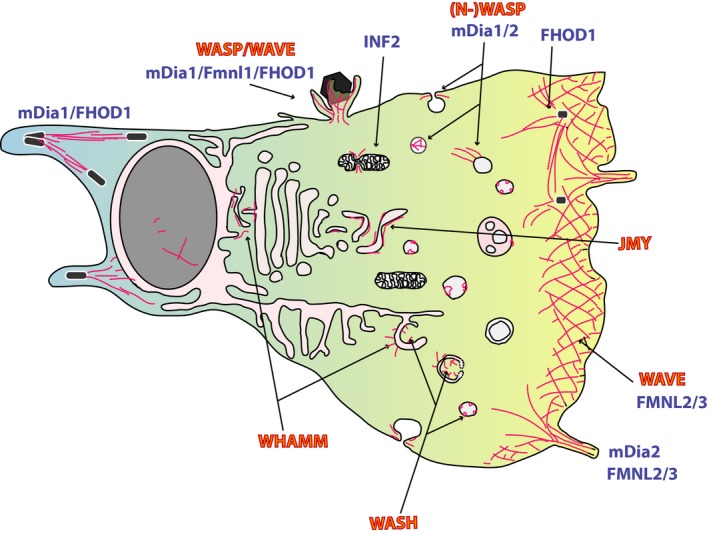
Cellular actin assemblies. Gross structure of cell membranes with actin assemblies and the respective Arp2/3‐complex activators (in red) or formins (in blue) that were described to contribute to their formation. The listing cannot be complete and requires continuous revision, as our knowledge on the cellular roles of these actin‐generating nanomachines is continuously growing. Original references for the mentioned actions of NPFs and formins are numerous and can be found in recent competitive reviews [2, 3, 4, 11, 13, 15]. Note that pathogens were found capable to usurp many if not most of these actin assemblies and that the currently unseen ones are expected to be found in the future.

The WASP family of NPFs in mammals now consists of four subgroups with eight members 4, namely Wasp/N‐WASP 5, 6, three WAVES 7, and the more recently identified WASH 8 and WHAMM/JMY 9, 10, 11 with individual cellular functions 12. As opposed to the Arp2/3 complex, the formin family, consisting of 15 members in mammals, generates long, unbranched filaments 13. Although certain formins are implicated in the formation of filopodia, which are finger‐shaped cell protrusions 14 or of myosin‐decorated stress fibers 13, sheet‐like protrusions termed lamellipodia embody the most prominent Arp2/3 complex‐mediated actin structure. Last but not least, consecutive copies of G‐actin‐binding domains, such as WH2 (WASP homology 2, also termed V domains for Verprolin homology domain) domains, are capable of generating filaments and represent an additional but in comparison still understudied class of actin nucleators 15. This class comprises members as different as Spire 16, Cobl (Cordon‐bleu, 17), leiomodin in muscle 18, 19, or the bacterial factors VopL and VopF from *Vibrio* sp. 20, 21. Finally, stability and turnover of actin filaments are controlled by a multitude of modulatory activities such as severing, capping or bundling, which determines, for example, texture, durability, or longevity of the given structure built. Together, we are still facing huge gaps in our understanding of how actin structures in living cells are formed through the concerted biochemical activities that we already know—aside from the unknown. A schematic overview of some actin‐nucleating gears and their preferred location of action—if known—are provided in Fig. 2.

### Rho GTPases signaling to actin assembly

Signaling pathways regulated by proteins of the Rho GTPase family are involved in many cellular functions, ranging from cell polarization, migration, cell division, and vesicle trafficking to transcription and inflammatory reactions, just to name a few 22.

**Figure 3:**
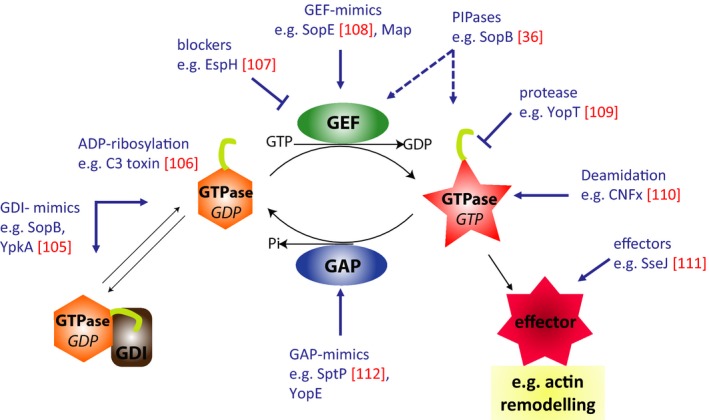
The Rho‐GTPase activation cycle and its manipulation by virulence factors. Rho GTPases cycle between an inactive GDP‐bound state and an active GTP‐bound state. So‐called GEFs regulate their activation, whereas GAPs enhance their intrinsic hydrolase leading to inactivation. In the GTP‐bound state, the GTPase binds to its downstream effectors. Finally, GDIs keep Rho GTPases in an inactive state and protect them from degradation. The small GTPase activation cycle is targeted by bacterial virulence factors at virtually every step. Virulence factors (in blue) are certainly not complete but just exemplary for entire families of factors and the identification of more virulence determinants and mechanisms is expected from future research. Targeting of these processes with small molecules might pave the way to novel pathoblockers or anticancer drugs.

Rho GTPases cycle between an inactive, GDP‐bound state and an active, GTP‐bound state. They undergo conformational changes during cycling between states, which in turn is controlled by other classes of GTPase‐binding proteins 23. So‐called guanine nucleotide exchange factors (GEFs, 24) regulate their activation by facilitating the exchange of GDP for GTP, whereas GTPase‐activating proteins (GAPs) enhance their intrinsic hydrolase activity leading to inactivation 25. In the GTP‐bound state, the GTPase binds to a given downstream effector, igniting a signaling cascade. Finally, guanine nucleotide dissociation inhibitors (GDIs) function to maintain Rho GTPases in an inactive GDP‐bound state 26 and/or protect them from degradation 27. The small GTPase activation cycle is schematically depicted in Fig. 3. The Rho GTPase family comprises 20 members in humans 28, with the best characterized members being RhoA, Rac1, and Cdc42.

RhoA has been shown to be involved in the formation of stress fibers, while Rac is responsible for the formation of actin‐rich protrusions termed lamellipodia. Cdc42 can instead contribute to the formation of various protrusions and to endomembrane trafficking, although it is still mostly associated with the formation of finger‐like filopodia. Owing to their conserved and crucial roles in controlling actin cytoskeleton turnover, cell survival, and proliferation, Rho GTPases are a prime target for virulence mechanisms of bacterial pathogens 29, 30, 31. It is worth mentioning here that bacterial virulence factors have evolved sophisticated examples of molecular mimicry, that is, harboring analogs of GTPase‐regulatory factors such as GEFs, GAPs, and GDIs (highlighted and referenced in Fig. 3).

## Actin structures induced or hijacked by bacteria

A subgroup of pathogenic bacteria invades their host cells such as nonphagocytic gut epithelium cells by stimulating uptake processes reminiscent of phagocytosis, macropinocytosis, or endocytosis. All these entry pathways converge on actin polymerization, although the phenotypic appearance is rather diverse. Historically, these invasion pathways were classified into so‐called ‘trigger’ and ‘zipper’ mechanisms 32, 33, either accompanied by excessive membrane ruffling mediated by large, lamellipodia‐like membrane folds, or alternatively, accompanied by much smaller, local actin rearrangements, respectively. Today, however, we know that this classification is not always as sharp between entry strategies of pathogens, and that bacteria can quite flexibly employ various entry pathways in different experimental systems that are not necessarily observed in their native target cells *in vivo*, which are usually much less accessible to experimental manipulation than established tissue culture models. Much work remains to be done in this area. Notwithstanding this, the virulence factors utilized and their molecular mechanisms of functions established in simplified, *in vitro* systems remain correct, although their output effects may be quantitatively and qualitatively different in cells of differentiated tissue.

For the trigger type of entry utilized for instance by *Shigella flexneri* or *Salmonella enterica* serovar Typhimurium, the pathogen transfers effector proteins into the host's cytoplasm (see T3SS below), inducing fierce, local actin polymerization, causing the plasma membrane to lift up and around the bacterium in order to envelop it. This is similar in appearance to the formation of phagocytic cups or large structures mediating macropinocytosis and engages virtually the same signaling and actin assemblies [34,35]. More recent research has uncovered, however, that pathogens can elicit many more and much more diverse responses in cells to induce their entry, engaging additional GTPases and actin‐dependent mechanisms unrelated to those initially identified, such as Rho‐mediated contractility 36 or SPIRE‐ and formin‐induced actin polymerization 37, 38.

The zipper mechanisms which are utilized, for example, by pathogenic *Yersinia and Listeria* species are initiated by bacterial surface proteins that serve as ‘fake’ ligands of host cell surface receptors. The receptor becomes activated and signals across the plasma membrane, which leads to highly localized actin polymerization events, reminiscent perhaps to those accompanying clathrin‐mediated endocytosis of the receptor. In the case of *Listeria*, two such mechanisms operate in parallel: one receptor‐ligand mimicry involves binding of bacterial Internalin A (InlA) to host E‐cadherin 39; the second mechanism concerns the c‐MET receptor tyrosine kinase binding to InlB 40, triggering of which during invasion of HeLa cells is accompanied by clathrin recruitment, supporting the idea of pathogen‐induced receptor endocytosis 41. In contrast, *Yersinia* utilizes the cell adhesion machinery through binding to the transmembrane protein β1‐integrin through the bacterial surface protein invasin 42.

### Bacterial virulence factors and Rho GTPases

A common virulence feature of gram‐negative gastrointestinal bacterial pathogens is the delivery of proteins directly into the host cell cytoplasm. The bacteria inject virulence factors, also known as effectors, via a syringe‐like nanomachine named Type III secretion system (T3SS), evolutionary related to the flagellum. While T3SSs are conserved in composition and function among different species, each bacterium secretes an individual set of effectors 43 thought to serve establishment of the individual niche. For instance, *Salmonella* and *Shigella* species are intracellular pathogens that trigger their uptake into nonphagocytic gut epithelial cells 44. Invasion into host cells of these bacteria depends on the activation of Rho GTPases by the concerted action of sets of T3 effectors that mediate prominent actin rearrangements resulting in engulfment of the bacteria 33. Quite distinct from those, members of the Enteropathogenic *E. coli* (EPEC)/Enterohemorrhagic *E. coli* (EHEC) group (also known as A/E lesion pathogens) are primarily extracellular, adhering to the surface of gut epithelial cells. Doing so, they induce loss of microvilli and induce formation of so‐called actin‐rich pedestals underneath their attachment points. These bacteria also deliver T3 effectors to manipulate the actin cytoskeleton 45.

In the last decade, work by Alto and colleagues was instrumental for the identification of a novel T3SS effector family, the WxxxE family of bacterial GEF mimics. Subsequent crystal structures revealed that WXXXE proteins in fact share the fold with Salmonella T3 effectors SopE/SopE2, also harboring GEF activity, and uncovered the elegant GEF mimicry mechanism 46, 47, 48.

In addition to these bacterial GEFs, also GAP and GDI mimics, or enzymes that modify GTPases for constitutive activation or inactivation exist, enabling manipulation of the host GTPase‐signaling landscape at various levels. All these factors have been described in comprehensive reviews 30, 49, 50 and some representative examples are given in Fig. 2.

Activation of specific individual Rho GTPases and corresponding actin‐generating machines engaged by these model pathogens were studied in detail over the past 20 years, but this has posed more questions than were answered. For instance, it is still in the dark how Rho is activated by the *Salmonella* phosphatidyl‐phosphate phosphatase SopB 36, or why *Shigella* harbors bacterial GEFs for the functionally antagonistic host GTPases Rac1 and RhoA 48, 51, 52 or how it recognizes tricellulin upon host contact 53, just to name a few. While quite some biochemical details on individual, bacterial virulence factors are now established, their intricate interplay—as they come as a cocktail—and a more holistic understanding of their profound effects in the host is galaxies away.

### Bacterial virulence factors and actin

The simplest mechanism of attacking the actin cytoskeleton is targeting it directly by modifying toxins, causing cross‐linking of actin or ADP‐ribosylation. These modifications either result in stimulation of actin polymerization or block it [reviewed in Ref. 54]. Bacterial virulence factors may also have modulatory functions such as actin bundling, as it was described for *Salmonella* SipA 55. Molecular mimicry of actin regulatory factors can occur at all levels (also compare Fig. 1): The *Listeria* surface protein ActA for instance mimics an NPF and recruits and activates Arp2/3 complex for actin tail formation. On the contrary, *Shigella* IcsA mimics an NPF‐activating signal and releases autoinhibition of the host cell NPF N‐WASP, which then recruits and activates Arp2/3 complex. These mechanisms lead to actin assembly at the bacterial surface in the cytoplasm followed host cell invasion. A further upstream type of mimicry is represented by Vaccinia Virus A36R or EPEC Tir, both of which mimic receptor tyrosine kinase (RTK) signaling through the plasma membrane 56. This leads to the recruitment of the RTK‐Adapters such as Nck, in turn igniting the N‐WASP‐Arp2/3 cascade and mediating actin tail formation at the plasma membrane abutting the pathogen upon clustering of the pathogenic receptor mimic. Alternative types of actin tail formation are exerted through bacterial actin nucleators like the Rickettsial protein Sca2 or the Burkholderial BimA, mimicking nucleation factors that generate long unbranched filaments with activities reminiscent of formins or Spire 57 or of the Ena/VASP family of actin polymerases 58. Remarkably, in case of BimA, different *Burkholderia* species have evolved this protein to either operate as Ena/VASP mimic (*B. pseudomallei* and *mallei*) or Arp2/3 complex activator (*B. thailandensis*), which confirmed the versatility and flexibility of virulence factor evolution to serve the specific pathogen's need 59. These and similar bacteria, residing and spreading inside host cells in an actin polymerization‐dependent fashion, have to exit the phagosome in order to unfold these features. Others like *Salmonella* remain in the membrane cover, and instead mature and remodel it to establish it as their specific niche. It is intuitive that this type of membrane remodeling will again involve Rho GTPases and actin dynamics, but the exact contributions of specific host cell factors are still in the dark.

## Actin and the viral life cycle

**Figure 4:**
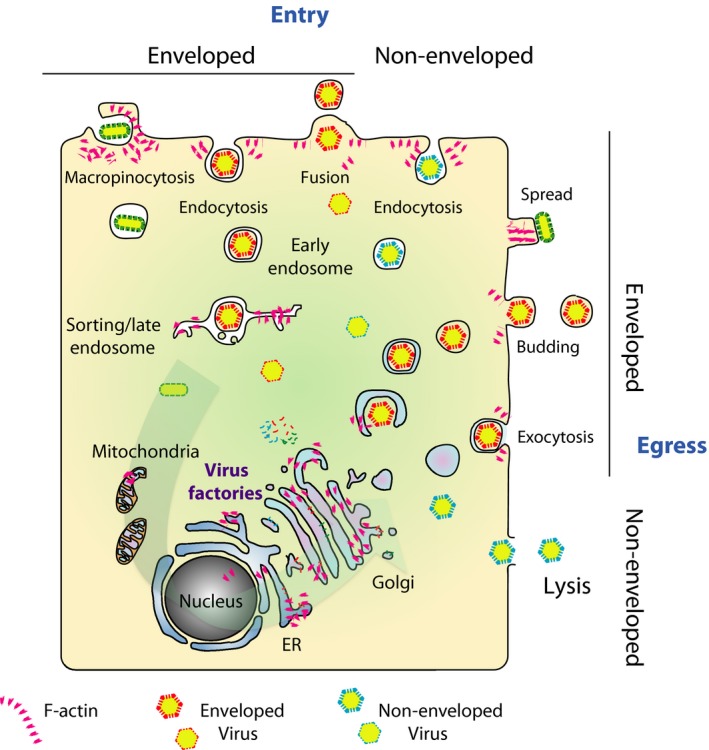
Virus infections harness actin assembly at membranes at all stages. Schematic representation of cellular locations where virus infection and propagation engages membranes and actin dynamics. The figure focuses on entry (upper side) and egress (right side) and only hints at the multiple possibilities of where virus assembly can take place such as ER and Golgi compartments. Virtually, every type of membrane and actin assembly is utilized by one or the other virus. Hence, it is not surprising that even mitochondria 98 or inhospitable places like peroxisomes can be exploited for virus propagation. Therefore, the figure must remain superficial and just repeats common themes. For instance, the term ‘endocytosis’ stands for all types of endocytosis not only clathrin‐mediated mechanisms.

Viruses depend as obligatory intracellular parasites on multiple functions of their host cell. Thus, viral infections unsurprisingly alter the regular functions of a cell to support replication and production of new virions. A prime aspect of this conversion is profound reorganization of the actin cytoskeleton, accompanying most if not all stages of the viral life cycle, from entry through replication and assembly to egress (Fig. 4) 60. One characteristic hallmark of viruses is their cellular and host tropism 61. In the absence of virus‐compatible host cells, they do not replicate at all. Two distinct subtypes of cellular viral tropism were described, namely receptor‐dependent and ‐independent tropisms. This means that restriction of viral replication occurs either on the cell surface (receptor‐dependent entry) or intracellularly (post‐entry steps) through molecular incompatibilities. The state of differentiation of a given cell dictates its gene expression pattern, which in turn enables (or prohibits) viral infection and propagation. Interestingly, several viruses can transform cells, which can be seen as an active step to design their new homes for persistence. This process also profoundly changes host cell proliferation and motility, often leading to tumor formation and metastasis. However, these processes will not be discussed here because it mostly is not an immediate form of host–pathogen interaction 62, 63. Nevertheless, it is worth to consider that these viruses apparently prefer to reside in motile and proliferating cells.

### Virus entry

In the first step of viral infection, virions engage the cell surface, subsequently penetrating the cell membrane and entering the cytoplasm.

Prior to internalization, many viruses show a cell‐surface‐surfing behavior, which is proposed to carry them from initial contact sites, for instance filopodial protrusions 64, to areas amendable for penetration into the cytoplasm, for example, sites with high‐endocytic activity. This process was shown to depend on actin and myosin II motor activity and likely be driven by myosin II‐dependent actin retrograde flow in these structures 65.

For subsequent internalization, the cortical actin meshwork is thought to embody a physical barrier that has to be overcome, which can be achieved by actin cytoskeleton remodeling 66. Virions can ignite signaling and induce internalization of their hijacked receptor, taking a ride on, for example, clathrin‐ or caveolin‐mediated endocytosis. Some virions utilize macropinocytosis or other clathrin‐independent paths into the cell, all involving actin in one of the other way [reviewed in Refs 67,68]. Apparently, viruses have learned to hijack the full spectrum of endocytic mechanisms to gain access to the cells.

Moreover, enveloped viruses such as HIV, HRSV, or HSV 69, 70, 71 may also gain entry by directly fusing with the cell's plasma membrane, which involves action of Rho GTPases and actin in a way that is not fully understood. Future research may identify correlates of this process in nonpathogenic cell fusion processes of the host, as found, for example, in muscle cell precursors or inflammatory macrophages forming giant cells.

Finally, attachment of virions to host cells may promote uptake of additional virions by stimulating signals rendering the host more susceptible. Herpes simplex virus (HSV), as an example, induces the extension of cell surface protrusions spiked with more attachment sites for more virions 72, 73.

### Intracellular trafficking, replication, assembly, and egress of virions

Dynamic actin turnover was shown to have strong effects on some viruses during their propagation in the host 74, 75, 76, 77. However, we are just beginning to distinguish the relative contributions of actin dynamics to these steps, using for instance super‐resolution video microscopy. On one hand, it is reasonable to assume that complex structures such as some virus factories in the cell center will strongly rely on intact actin dynamics to support rearrangements of ER and Golgi in response to virion production. However, there is much more to be explored in this phase of the viral life cycle: actin impacts on eukaryotic gene expression directly 78, 79 and indirectly 80, 81 and, in addition, contributes to chromatin organization through nuclear F‐actin assemblies, but how precisely remains to be established 82. Although these aspects of actin dynamics are incompletely understood and notoriously difficult to visualize, even less is known about usurpation of them by virions. Nevertheless, several indications for the participation of these cellular processes in virion production/assembly have emerged 83, 84, 85, 86, 87, 88.

Lately, we have witnessed an explosion of knowledge on autophagy. Autophagy comes in various flavors in the cell, but is accompanied by distinct membrane remodeling events that all involve actin dynamics 89 mostly downstream of Arp2/3 complex‐dependent and the corresponding NPFs WASH, WHAMM, and JMY 90, 91, 92, 93. Not surprisingly, therefore, this cellular process is also connected to the life cycle of various viruses. Although some have evolved to evade autophagy in the cell, others appear to have modified autophagy for their own benefit. However, the connection between actin dynamics, autophagy, and viral infection is still comparably vague and I would like to refer to two excellent recent reviews summarizing this emerging field 94, 95. Future research will have to define whether virions directly target actin dynamics during manipulation of the autophagic flux, or if this connection is indirect.

Next, budding and egress steps of viral pathogens again involve passing through the plasma membrane, which necessarily requires actin rearrangements. It is known, for instance, that some viruses including HIV induce actin‐based protrusions/microvilli 96 and that actin depolymerization diminishes viral yield. Moreover, cell to cell spread of this virus involving the viral Env and GAG proteins is actin‐dependent, and indeed, HIV‐GAG directly interacts with F‐actin 97.

Finally, virus spread may also be promoted by direct induction of actin structures. As a prominent example, vaccinia virus and other members of the poxvirus family are well known for inducing prominent actin structures below the plasma membrane following budding, again generating actin comet tails now considered important for efficient viral dissemination. Comparable structures are induced at the cell surface through signaling across the plasma membrane by pathogenic *Escherichia coli*, for instance of the EPEC or EHEC type (see above and Ref. 31). Although certainly more static than Vaccinia virus tails (see above) and thus specifically called actin pedestals, these structures are believed to mediate translocation of the bacteria along the plasma membrane and perhaps onto neighboring cells. This emphasizes how the same pathways and machineries can lead to distinct output responses, which must depend on the overall molecular inventory of host cell proteins regulating these pathogen‐induced actin structures.

Together, due to the intimate contact and obligate dependence of the virus on the host cell equipment, coevolution has shaped a multitude of strategies that all either directly utilize manipulation of actin (dis‐)assembly or at least take into account that the targeted membrane is under control of actin dynamics. Future work needs to dissect the differential contribution of signaling and actin assembly factors to the steps of individual viral life cycles (Fig. 4).

## Concluding remarks

All intracellular and even some extracellular pathogens subvert the host cell cytoskeleton to promote their own survival, replication, and dissemination. A study of these microbes has led to important discoveries concerning not only the specific infection mechanism at play but also regarding the specific function of cytoskeletal regulatory pathways and cellular mechanisms. Importantly, the cellular pathways involved may harbor attractive therapeutic targets to fight such infections. However, to reach this goal, much work is required to tease apart ‘bystanders’, recruitment of which just accompanies these processes, from ‘drivers’, directly utilized by the pathogen, which might embody promising targets. Aim of such approaches is not necessarily to kill the microbe, which would pose a selection pressure to developing further resistances, but to tune down the dynamics of a given infection allowing the host to eradicate the intruder by itself. Novel systematic analyses, including systems biology level comprehension of these processes and molecular biology down to atomic resolution, are required to enlighten the delicate interaction processes between pathogen and host.

## Author contributions

TS drafted the manuscript and drew the figures. TS and MS wrote the manuscript.

## References

[1] Rottner K and Stradal TE (2011) Actin dynamics and turnover in cell motility. Curr Opin Cell Biol 23, 569–578. 21807492 10.1016/j.ceb.2011.07.003

[2] Rottner K , Faix J , Bogdan S , Linder S and Kerkhoff E (2017) Actin assembly mechanisms at a glance. J Cell Sci 130, 3427–3435. 29032357 10.1242/jcs.206433

[3] Siton‐Mendelson O and Bernheim‐Groswasser A (2017) Functional actin networks under construction: the cooperative action of actin nucleation and elongation factors. Trends Biochem Sci 42, 414–430. 28372857 10.1016/j.tibs.2017.03.002

[4] Alekhina O , Burstein E and Billadeau DD (2017) Cellular functions of WASP family proteins at a glance. J Cell Sci 130, 2235–2241. 28646090 10.1242/jcs.199570 PMC5536917

[5] Derry JM , Ochs HD and Francke U (1994) Isolation of a novel gene mutated in Wiskott‐Aldrich syndrome. Cell 78, 635–644. 8069912 10.1016/0092-8674(94)90528-2

[6] Miki H , Miura K and Takenawa T (1996) N‐WASP, a novel actin‐depolymerizing protein, regulates the cortical cytoskeletal rearrangement in a PIP2‐dependent manner downstream of tyrosine kinases. EMBO J 15, 5326–5335. 8895577 PMC452276

[7] Suetsugu S , Miki H and Takenawa T (1999) Identification of two human WAVE/SCAR homologues as general actin regulatory molecules which associate with the Arp2/3 complex. Biochem Biophys Res Commun 260, 296–302. 10381382 10.1006/bbrc.1999.0894

[8] Linardopoulou EV , Parghi SS , Friedman C , Osborn GE , Parkhurst SM and Trask BJ (2007) Human subtelomeric WASH genes encode a new subclass of the WASP family. PLoS Genet 3, e237. 18159949 10.1371/journal.pgen.0030237 PMC2151093

[9] Zuchero JB , Coutts AS , Quinlan ME , Thangue NB and Mullins RD (2009) p53‐cofactor JMY is a multifunctional actin nucleation factor. Nat Cell Biol 11, 451–459. 19287377 10.1038/ncb1852 PMC2763628

[10] Campellone KG , Webb NJ , Znameroski EA and Welch MD (2008) WHAMM is an Arp2/3 complex activator that binds microtubules and functions in ER to Golgi transport. Cell 134, 148–161. 18614018 10.1016/j.cell.2008.05.032 PMC2556884

[11] Rottner K , Hanisch J and Campellone KG (2010) WASH, WHAMM and JMY: regulation of Arp2/3 complex and beyond. Trends Cell Biol 20, 650–661. 20888769 10.1016/j.tcb.2010.08.014

[12] Steffen A , Stradal TE and Rottner K (2017) Signalling pathways controlling cellular actin organization. Handb Exp Pharmacol 235, 153–178. 27757765 10.1007/164_2016_35

[13] Kuhn S and Geyer M (2014) Formins as effector proteins of Rho GTPases. Small GTPases 5, e29513. 24914801 10.4161/sgtp.29513 PMC4111664

[14] Faix J , Breitsprecher D , Stradal TE and Rottner K (2009) Filopodia: complex models for simple rods. Int J Biochem Cell Biol 41, 1656–1664. 19433307 10.1016/j.biocel.2009.02.012

[15] Dominguez R (2016) The WH2 domain and actin nucleation: necessary but insufficient. Trends Biochem Sci 41, 478–490. 27068179 10.1016/j.tibs.2016.03.004 PMC4884163

[16] Quinlan ME , Heuser JE , Kerkhoff E and Mullins RD (2005) Drosophila spire is an actin nucleation factor. Nature 433, 382–388. 15674283 10.1038/nature03241

[17] Ahuja R , Pinyol R , Reichenbach N , Custer L , Klingensmith J , Kessels MM and Qualmann B (2007) Cordon‐bleu is an actin nucleation factor and controls neuronal morphology. Cell 131, 337–350. 17956734 10.1016/j.cell.2007.08.030 PMC2507594

[18] Chereau D , Boczkowska M , Skwarek‐Maruszewska A , Fujiwara I , Hayes DB , Rebowski G , Lappalainen P , Pollard TD and Dominguez R (2008) Leiomodin is an actin filament nucleator in muscle cells. Science 320, 239–243. 18403713 10.1126/science.1155313 PMC2845909

[19] Chen X , Ni F , Kondrashkina E , Ma J and Wang Q (2015) Mechanisms of leiomodin 2‐mediated regulation of actin filament in muscle cells. Proc Natl Acad Sci U S A 112, 12687–12692. 26417072 10.1073/pnas.1512464112 PMC4611611

[20] Burke TA , Harker AJ , Dominguez R and Kovar DR (2017) The bacterial virulence factors VopL and VopF nucleate actin from the pointed end. J Cell Biol 216, 1267–1276. 28363971 10.1083/jcb.201608104 PMC5412564

[21] Tam VC , Suzuki M , Coughlin M , Saslowsky D , Biswas K , Lencer WI , Faruque SM and Mekalanos JJ (2010) Functional analysis of VopF activity required for colonization in *Vibrio cholerae* . MBio 1, e00289‐10. 21151774 10.1128/mBio.00289-10 PMC2999938

[22] Hall A (2012) Rho family GTPases. Biochem Soc Trans 40, 1378–1382. 23176484 10.1042/BST20120103

[23] Corbett KD and Alber T (2001) The many faces of Ras: recognition of small GTP‐binding proteins. Trends Biochem Sci 26, 710–716. 11738594 10.1016/s0968-0004(01)01974-0

[24] Rossman KL , Der CJ and Sondek J (2005) GEF means go: turning on RHO GTPases with guanine nucleotide‐exchange factors. Nat Rev Mol Cell Biol 6, 167–180. 15688002 10.1038/nrm1587

[25] Vetter IR and Wittinghofer A (2001) The guanine nucleotide‐binding switch in three dimensions. Science 294, 1299–1304. 11701921 10.1126/science.1062023

[26] Scheffzek K , Stephan I , Jensen ON , Illenberger D and Gierschik P (2000) The Rac‐RhoGDI complex and the structural basis for the regulation of Rho proteins by RhoGDI. Nat Struct Biol 7, 122–126. 10655614 10.1038/72392

[27] Boulter E , Garcia‐Mata R , Guilluy C , Dubash A , Rossi G , Brennwald PJ and Burridge K (2010) Regulation of Rho GTPase crosstalk, degradation and activity by RhoGDI1. Nat Cell Biol 12, 477–483. 20400958 10.1038/ncb2049 PMC2866742

[28] Heasman SJ and Ridley AJ (2008) Mammalian Rho GTPases: new insights into their functions from in vivo studies. Nat Rev Mol Cell Biol 9, 690–701. 18719708 10.1038/nrm2476

[29] Finlay BB (2005) Bacterial virulence strategies that utilize Rho GTPases. Curr Top Microbiol Immunol 291, 1–10. 15981456 10.1007/3-540-27511-8_1

[30] Popoff MR (2014) Bacterial factors exploit eukaryotic Rho GTPase signaling cascades to promote invasion and proliferation within their host. Small GTPases 5, e983863. 10.4161/sgtp.28209 PMC4160336 25203748

[31] Stradal TE and Costa SC (2017) Type III secreted virulence factors manipulating signaling to actin dynamics. Curr Top Microbiol Immunol 399, 175–199. 27744505 10.1007/82_2016_35

[32] Cossart P and Sansonetti PJ (2004) Bacterial invasion: the paradigms of enteroinvasive pathogens. Science 304, 242–248. 15073367 10.1126/science.1090124

[33] Rottner K , Stradal TE and Wehland J (2005) Bacteria‐host‐cell interactions at the plasma membrane: stories on actin cytoskeleton subversion. Dev Cell 9, 3–17. 15992537 10.1016/j.devcel.2005.06.002

[34] Loh LN , McCarthy EMC , Narang P , Khan NA and Ward TH (2017) *Escherichia coli* K1 utilizes host macropinocytic pathways for invasion of brain microvascular endothelial cells. Traffic 18, 733–746. 28799243 10.1111/tra.12508

[35] Chen LM , Hobbie S and Galan JE (1996) Requirement of CDC42 for *Salmonella*‐induced cytoskeletal and nuclear responses. Science 274, 2115–2118. 8953049 10.1126/science.274.5295.2115

[36] Hanisch J , Kolm R , Wozniczka M , Bumann D , Rottner K and Stradal TE (2011) Activation of a RhoA/Myosin II‐dependent but Arp2/3 complex‐independent pathway facilitates *Salmonella* invasion. Cell Host Microbe 9, 273–285. 21501827 10.1016/j.chom.2011.03.009

[37] Andritschke D , Dilling S , Emmenlauer M , Welz T , Schmich F , Misselwitz B , Ramo P , Rottner K , Kerkhoff E , Wada T *et al* (2016) A genome‐wide siRNA screen implicates Spire1/2 in SipA‐driven Salmonella Typhimurium host cell invasion. PLoS One 11, e0161965. 27627128 10.1371/journal.pone.0161965 PMC5023170

[38] Truong D , Brabant D , Bashkurov M , Wan LC , Braun V , Heo WD , Meyer T , Pelletier L , Copeland J and Brumell JH (2013) Formin‐mediated actin polymerization promotes *Salmonella* invasion. Cell Microbiol 15, 2051–2063. 23869992 10.1111/cmi.12173

[39] Mengaud J , Ohayon H , Gounon P , Mege RM and Cossart P (1996) E‐cadherin is the receptor for internalin, a surface protein required for entry of *L. monocytogenes* into epithelial cells. Cell 84, 923–932. 8601315 10.1016/s0092-8674(00)81070-3

[40] Shen Y , Naujokas M , Park M and Ireton K (2000) InIB‐dependent internalization of *Listeria* is mediated by the Met receptor tyrosine kinase. Cell 103, 501–510. 11081636 10.1016/s0092-8674(00)00141-0

[41] Veiga E and Cossart P (2005) Listeria hijacks the clathrin‐dependent endocytic machinery to invade mammalian cells. Nat Cell Biol 7, 894–900. 16113677 10.1038/ncb1292

[42] Isberg RR and Leong JM (1990) Multiple beta 1 chain integrins are receptors for invasin, a protein that promotes bacterial penetration into mammalian cells. Cell 60, 861–871. 2311122 10.1016/0092-8674(90)90099-z

[43] Galan JE , Lara‐Tejero M , Marlovits TC and Wagner S (2014) Bacterial type III secretion systems: specialized nanomachines for protein delivery into target cells. Annu Rev Microbiol 68, 415–438. 25002086 10.1146/annurev-micro-092412-155725 PMC4388319

[44] Dunn JD and Valdivia RH (2010) Uncivil engineers: *Chlamydia*,* Salmonella* and *Shigella* alter cytoskeleton architecture to invade epithelial cells. Future Microbiol 5, 1219–1232. 20722600 10.2217/fmb.10.77

[45] Campellone KG (2010) Cytoskeleton‐modulating effectors of enteropathogenic and enterohaemorrhagic *Escherichia coli*: Tir, EspFU and actin pedestal assembly. FEBS J 277, 2390–2402. 20477869 10.1111/j.1742-4658.2010.07653.x

[46] Buchwald G , Friebel A , Galan JE , Hardt WD , Wittinghofer A and Scheffzek K (2002) Structural basis for the reversible activation of a Rho protein by the bacterial toxin SopE. EMBO J 21, 3286–3295. 12093730 10.1093/emboj/cdf329 PMC126081

[47] Huang Z , Sutton SE , Wallenfang AJ , Orchard RC , Wu X , Feng Y , Chai J and Alto NM (2009) Structural insights into host GTPase isoform selection by a family of bacterial GEF mimics. Nat Struct Mol Biol 16, 853–860. 19620963 10.1038/nsmb.1647 PMC5130228

[48] Klink BU , Barden S , Heidler TV , Borchers C , Ladwein M , Stradal TE , Rottner K and Heinz DW (2010) Structure of Shigella IpgB2 in complex with human RhoA: implications for the mechanism of bacterial guanine nucleotide exchange factor mimicry. J Biol Chem 285, 17197–17208. 20363740 10.1074/jbc.M110.107953 PMC2878080

[49] Aktories K (2011) Bacterial protein toxins that modify host regulatory GTPases. Nat Rev Microbiol 9, 487–498. 21677684 10.1038/nrmicro2592

[50] Lemichez E and Aktories K (2013) Hijacking of Rho GTPases during bacterial infection. Exp Cell Res 319, 2329–2336. 23648569 10.1016/j.yexcr.2013.04.021

[51] Handa Y , Suzuki M , Ohya K , Iwai H , Ishijima N , Koleske AJ , Fukui Y and Sasakawa C (2007) Shigella IpgB1 promotes bacterial entry through the ELMO‐Dock180 machinery. Nat Cell Biol 9, 121–128. 17173036 10.1038/ncb1526

[52] Hachani A , Biskri L , Rossi G , Marty A , Menard R , Sansonetti P , Parsot C , Van Nhieu GT , Bernardini ML and Allaoui A (2008) IpgB1 and IpgB2, two homologous effectors secreted via the Mxi‐Spa type III secretion apparatus, cooperate to mediate polarized cell invasion and inflammatory potential of *Shigella flexenri* . Microbes Infect 10, 260–268. 18316224 10.1016/j.micinf.2007.11.011

[53] Fukumatsu M , Ogawa M , Arakawa S , Suzuki M , Nakayama K , Shimizu S , Kim M , Mimuro H and Sasakawa C (2012) Shigella targets epithelial tricellular junctions and uses a noncanonical clathrin‐dependent endocytic pathway to spread between cells. Cell Host Microbe 11, 325–336. 22520461 10.1016/j.chom.2012.03.001

[54] Aktories K , Schwan C and Lang AE (2017) ADP‐ribosylation and cross‐linking of actin by bacterial protein toxins. Handb Exp Pharmacol 235, 179–206. 27316913 10.1007/164_2016_26

[55] McGhie EJ , Hayward RD and Koronakis V (2004) Control of actin turnover by a salmonella invasion protein. Mol Cell 13, 497–510. 14992720 10.1016/s1097-2765(04)00053-x

[56] Frischknecht F , Moreau V , Rottger S , Gonfloni S , Reckmann I , Superti‐Furga G and Way M (1999) Actin‐based motility of vaccinia virus mimics receptor tyrosine kinase signalling. Nature 401, 926–929. 10553910 10.1038/44860

[57] Haglund CM , Choe JE , Skau CT , Kovar DR and Welch MD (2010) Rickettsia Sca2 is a bacterial formin‐like mediator of actin‐based motility. Nat Cell Biol 12, 1057–1063. 20972427 10.1038/ncb2109 PMC3136050

[58] Benanti EL , Nguyen CM and Welch MD (2015) Virulent *Burkholderia* species mimic host actin polymerases to drive actin‐based motility. Cell 161, 348–360. 25860613 10.1016/j.cell.2015.02.044 PMC4393530

[59] Bugalhao JN , Mota LJ and Franco IS (2015) Bacterial nucleators: actin’ on actin. Pathog Dis 73, ftv078. 26416078 10.1093/femspd/ftv078 PMC4626583

[60] Taylor MP , Koyuncu OO and Enquist LW (2011) Subversion of the actin cytoskeleton during viral infection. Nat Rev Microbiol 9, 427–439. 21522191 10.1038/nrmicro2574 PMC3229036

[61] Nomaguchi M , Fujita M , Miyazaki Y and Adachi A (2012) Viral tropism. Front Microbiol 3, 281. 22876241 10.3389/fmicb.2012.00281 PMC3411105

[62] Litwin TR , Clarke MA , Dean M and Wentzensen N (2017) Somatic host cell alterations in HPV carcinogenesis. Viruses 9, 206. 10.3390/v9080206 PMC5580463 28771191

[63] Valderrama F , Cordeiro JV , Schleich S , Frischknecht F and Way M (2006) Vaccinia virus‐induced cell motility requires F11L‐mediated inhibition of RhoA signaling. Science 311, 377–381. 16424340 10.1126/science.1122411

[64] Lehmann MJ , Sherer NM , Marks CB , Pypaert M and Mothes W (2005) Actin‐ and myosin‐driven movement of viruses along filopodia precedes their entry into cells. J Cell Biol 170, 317–325. 16027225 10.1083/jcb.200503059 PMC2171413

[65] Medeiros NA , Burnette DT and Forscher P (2006) Myosin II functions in actin‐bundle turnover in neuronal growth cones. Nat Cell Biol 8, 215–226. 16501565 10.1038/ncb1367

[66] Kizhatil K and Albritton LM (1997) Requirements for different components of the host cell cytoskeleton distinguish ecotropic murine leukemia virus entry via endocytosis from entry via surface fusion. J Virol 71, 7145–7156. 9311787 10.1128/jvi.71.10.7145-7156.1997 PMC192054

[67] Doherty GJ and McMahon HT (2009) Mechanisms of endocytosis. Annu Rev Biochem 78, 857–902. 19317650 10.1146/annurev.biochem.78.081307.110540

[68] Mooren OL , Galletta BJ and Cooper JA (2012) Roles for actin assembly in endocytosis. Annu Rev Biochem 81, 661–686. 22663081 10.1146/annurev-biochem-060910-094416

[69] Eisenberg RJ , Atanasiu D , Cairns TM , Gallagher JR , Krummenacher C and Cohen GH (2012) Herpes virus fusion and entry: a story with many characters. Viruses 4, 800–832. 22754650 10.3390/v4050800 PMC3386629

[70] Klasse PJ (2012) The molecular basis of HIV entry. Cell Microbiol 14, 1183–1192. 22583677 10.1111/j.1462-5822.2012.01812.x PMC3417324

[71] Cox RG and Williams JV (2013) Breaking in: human metapneumovirus fusion and entry. Viruses 5, 192–210. 23325326 10.3390/v5010192 PMC3564117

[72] Clement C , Tiwari V , Scanlan PM , Valyi‐Nagy T , Yue BY and Shukla D (2006) A novel role for phagocytosis‐like uptake in herpes simplex virus entry. J Cell Biol 174, 1009–1021. 17000878 10.1083/jcb.200509155 PMC2064392

[73] Smith JL , Lidke DS and Ozbun MA (2008) Virus activated filopodia promote human papillomavirus type 31 uptake from the extracellular matrix. Virology 381, 16–21. 18834609 10.1016/j.virol.2008.08.040 PMC3253369

[74] Kallewaard NL , Bowen AL and Crowe JE Jr (2005) Cooperativity of actin and microtubule elements during replication of respiratory syncytial virus. Virology 331, 73–81. 15582654 10.1016/j.virol.2004.10.010

[75] Burke E , Mahoney NM , Almo SC and Barik S (2000) Profilin is required for optimal actin‐dependent transcription of respiratory syncytial virus genome RNA. J Virol 74, 669–675. 10623728 10.1128/jvi.74.2.669-675.2000 PMC111586

[76] Harpen M , Barik T , Musiyenko A and Barik S (2009) Mutational analysis reveals a noncontractile but interactive role of actin and profilin in viral RNA‐dependent RNA synthesis. J Virol 83, 10869–10876. 19710142 10.1128/JVI.01271-09 PMC2772787

[77] Fackler OT and Krausslich HG (2006) Interactions of human retroviruses with the host cell cytoskeleton. Curr Opin Microbiol 9, 409–415. 16820319 10.1016/j.mib.2006.06.010

[78] Zheng B , Han M , Bernier M and Wen JK (2009) Nuclear actin and actin‐binding proteins in the regulation of transcription and gene expression. FEBS J 276, 2669–2685. 19459931 10.1111/j.1742-4658.2009.06986.x PMC2978034

[79] Viita T and Vartiainen MK (2017) From cytoskeleton to gene expression: actin in the nucleus. Handb Exp Pharmacol 235, 311–329. 27316910 10.1007/164_2016_27

[80] Posern G and Treisman R (2006) Actin’ together: serum response factor, its cofactors and the link to signal transduction. Trends Cell Biol 16, 588–596. 17035020 10.1016/j.tcb.2006.09.008

[81] Olson EN and Nordheim A (2010) Linking actin dynamics and gene transcription to drive cellular motile functions. Nat Rev Mol Cell Biol 11, 353–365. 20414257 10.1038/nrm2890 PMC3073350

[82] Baarlink C , Plessner M , Sherrard A , Morita K , Misu S , Virant D , Kleinschnitz EM , Harniman R , Alibhai D , Baumeister S *et al* (2017) A transient pool of nuclear F‐actin at mitotic exit controls chromatin organization. Nat Cell Biol 19, 1389–1399. 29131140 10.1038/ncb3641

[83] Marek M , Merten OW , Galibert L , Vlak JM and van Oers MM (2011) Baculovirus VP80 protein and the F‐actin cytoskeleton interact and connect the viral replication factory with the nuclear periphery. J Virol 85, 5350–5362. 21450830 10.1128/JVI.00035-11 PMC3094977

[84] Bukrinskaya A , Brichacek B , Mann A and Stevenson M (1998) Establishment of a functional human immunodeficiency virus type 1 (HIV‐1) reverse transcription complex involves the cytoskeleton. J Exp Med 188, 2113–2125. 9841925 10.1084/jem.188.11.2113 PMC2212381

[85] Wilkie AR , Lawler JL and Coen DM (2016) A role for nuclear F‐Actin induction in human cytomegalovirus nuclear egress. MBio 7, e01254‐16. 27555312 10.1128/mBio.01254-16 PMC4999551

[86] Katsch K , de Jong SJ , Albrecht JC , Steger J , Genth H , Posern G and Biesinger B (2012) Actin‐dependent activation of serum response factor in T cells by the viral oncoprotein tip. Cell Commun Signal 10, 5. 22385615 10.1186/1478-811X-10-5 PMC3310822

[87] Bosse JB , Hogue IB , Feric M , Thiberge SY , Sodeik B , Brangwynne CP and Enquist LW (2015) Remodeling nuclear architecture allows efficient transport of herpesvirus capsids by diffusion. Proc Natl Acad Sci U S A 112, E5725–E5733. 26438852 10.1073/pnas.1513876112 PMC4620878

[88] Forest T , Barnard S and Baines JD (2005) Active intranuclear movement of herpesvirus capsids. Nat Cell Biol 7, 429–431. 15803134 10.1038/ncb1243

[89] Zientara‐Rytter K and Subramani S (2016) Role of actin in shaping autophagosomes. Autophagy 12, 2512–2515. 27723370 10.1080/15548627.2016.1236877 PMC5173263

[90] King JS , Gueho A , Hagedorn M , Gopaldass N , Leuba F , Soldati T and Insall RH (2013) WASH is required for lysosomal recycling and efficient autophagic and phagocytic digestion. Mol Biol Cell 24, 2714–2726. 23885127 10.1091/mbc.E13-02-0092 PMC3756923

[91] Xia P , Wang S , Du Y , Zhao Z , Shi L , Sun L , Huang G , Ye B , Li C , Dai Z *et al* (2013) WASH inhibits autophagy through suppression of Beclin 1 ubiquitination. EMBO J 32, 2685–2696. 23974797 10.1038/emboj.2013.189 PMC3801434

[92] Kast DJ , Zajac AL , Holzbaur EL , Ostap EM and Dominguez R (2015) WHAMM directs the Arp2/3 complex to the ER for autophagosome biogenesis through an actin comet tail mechanism. Curr Biol 25, 1791–1797. 26096974 10.1016/j.cub.2015.05.042 PMC4489997

[93] Coutts AS and La Thangue NB (2015) Actin nucleation by WH2 domains at the autophagosome. Nat Commun 6, 7888. 26223951 10.1038/ncomms8888 PMC4532831

[94] Abdoli A , Alirezaei M , Mehrbod P and Forouzanfar F (2018) Autophagy: the multi‐purpose bridge in viral infections and host cells. Rev Med Virol, e1973. 29709097 10.1002/rmv.1973 PMC7169200

[95] Viret C , Rozieres A and Faure M (2018) Autophagy during early virus‐host cell interactions. J Mol Biol 430, 1696–1713. 29698649 10.1016/j.jmb.2018.04.018

[96] Carlson LA , de Marco A , Oberwinkler H , Habermann A , Briggs JA , Krausslich HG and Grunewald K (2010) Cryo electron tomography of native HIV‐1 budding sites. PLoS Pathog 6, e1001173. 21124872 10.1371/journal.ppat.1001173 PMC2991257

[97] Chen C , Jin J , Rubin M , Huang L , Sturgeon T , Weixel KM , Stolz DB , Watkins SC , Bamburg JR , Weisz OA *et al* (2007) Association of gag multimers with filamentous actin during equine infectious anemia virus assembly. Curr HIV Res 5, 315–323. 17504173 10.2174/157016207780636542

[98] Mezeth KB , Nylund S , Henriksen H , Patel S , Nerland AH and Szilvay AM (2007) RNA‐dependent RNA polymerase from Atlantic halibut nodavirus contains two signals for localization to the mitochondria. Virus Res 130, 43–52. 17602779 10.1016/j.virusres.2007.05.014

[99] Tezcan‐Merdol D , Nyman T , Lindberg U , Haag F , Koch‐Nolte F and Rhen M (2001) Actin is ADP‐ribosylated by the *Salmonella enterica* virulence‐associated protein SpvB. Mol Microbiol 39, 606–619. 11169102 10.1046/j.1365-2958.2001.02258.x

[100] Welch MD , Rosenblatt J , Skoble J , Portnoy DA and Mitchison TJ (1998) Interaction of human Arp2/3 complex and the *Listeria monocytogenes* ActA protein in actin filament nucleation. Science 281, 105–108. 9651243 10.1126/science.281.5373.105

[101] Egile C , Loisel TP , Laurent V , Li R , Pantaloni D , Sansonetti PJ and Carlier MF (1999) Activation of the CDC42 effector N‐WASP by the *Shigella flexneri* IcsA protein promotes actin nucleation by Arp2/3 complex and bacterial actin‐based motility. J Cell Biol 146, 1319–1332. 10491394 10.1083/jcb.146.6.1319 PMC2156126

[102] Liverman AD , Cheng HC , Trosky JE , Leung DW , Yarbrough ML , Burdette DL , Rosen MK and Orth K (2007) Arp2/3‐independent assembly of actin by Vibrio type III effector VopL. Proc Natl Acad Sci U S A 104, 17117–17122. 17942696 10.1073/pnas.0703196104 PMC2040399

[103] Hayward RD and Koronakis V (1999) Direct nucleation and bundling of actin by the SipC protein of invasive *Salmonella* . EMBO J 18, 4926–4934. 10487745 10.1093/emboj/18.18.4926 PMC1171564

[104] McGhie EJ , Hayward RD and Koronakis V (2001) Cooperation between actin‐binding proteins of invasive *Salmonella*: SipA potentiates SipC nucleation and bundling of actin. EMBO J 20, 2131–2139. 11331579 10.1093/emboj/20.9.2131 PMC125241

[105] Prehna G , Ivanov MI , Bliska JB and Stebbins CE (2006) Yersinia virulence depends on mimicry of host Rho‐family nucleotide dissociation inhibitors. Cell 126, 869–880. 16959567 10.1016/j.cell.2006.06.056

[106] Braun U , Habermann B , Just I , Aktories K and Vandekerckhove J (1989) Purification of the 22 kDa protein substrate of botulinum ADP‐ribosyltransferase C3 from porcine brain cytosol and its characterization as a GTP‐binding protein highly homologous to the rho gene product. FEBS Lett 243, 70–76. 2493391 10.1016/0014-5793(89)81220-7

[107] Dong N , Liu L and Shao F (2010) A bacterial effector targets host DH‐PH domain RhoGEFs and antagonizes macrophage phagocytosis. EMBO J 29, 1363–1376. 20300064 10.1038/emboj.2010.33 PMC2868573

[108] Hardt WD , Chen LM , Schuebel KE , Bustelo XR and Galan JE (1998) S. typhimurium encodes an activator of Rho GTPases that induces membrane ruffling and nuclear responses in host cells. Cell 93, 815–826. 9630225 10.1016/s0092-8674(00)81442-7

[109] Shao F and Dixon JE (2003) YopT is a cysteine protease cleaving Rho family GTPases. Adv Exp Med Biol 529, 79–84. 12756732 10.1007/0-306-48416-1_14

[110] Knust Z and Schmidt G (2010) Cytotoxic necrotizing factors (CNFs) ‐ a growing toxin family. Toxins (Basel) 2, 116–127. 22069550 10.3390/toxins2010116 PMC3206620

[111] Christen M , Coye LH , Hontz JS , LaRock DL , Pfuetzner RA , Megha and Miller SI (2009) Activation of a bacterial virulence protein by the GTPase RhoA. Sci Signal 2, ra71. 19887681 10.1126/scisignal.2000430 PMC5096451

[112] Fu Y and Galan JE (1999) A salmonella protein antagonizes Rac‐1 and Cdc42 to mediate host‐cell recovery after bacterial invasion. Nature 401, 293–297. 10499590 10.1038/45829

